# Phenacetin in the Treatment of Neuralgia

**Published:** 1889-09

**Authors:** 


### Phenacetin in the Treatment of Neuralgia.

According to Dr. Ott (*Zeitschrift für Therapie*, May 15, 1889) phenacetin, in doses of from seven and one-half to seventy-five grains daily, possesses most marked value, according to his experience, in the treatment of neuralgia of peripheral origin; while it is without action in the treatment of neuralgia dependent upon disease of the brain or spinal cord. Dr. Ott administers the remedy in the form of a powder enclosed in capsules, in doses of seven and one-half grains, and has never had occasion to use larger amounts. One or two of these powders, given at intervals of an hour, are found to succeed easily in arresting suffering. His most brilliant results are stated to have been obtained through the use of phenacetin in hemicrania, and in occipital neuralgia, which so frequently occurs in women during the menstrual period, or in men as a consequence of marked hemorrhoidal congestion. In one case of hemicrania he had marked success even after the patient had taken antifebrin without avail. In pure trigeminal neuralgia it only produces transient relief, so that final resort must be had to other remedies, such as quinine, salicyl, or electricity. He reports two cases of cardialgia in which there was marked relief, as well as three cases of intercostal neuralgia. On the other hand, it produced no effect in an extremely severe case of sciatica, even though the dose was increased to seventy-five grains in twenty-four hours. Unfortunately, the economy appears to be accustomed to the use of phenacetin, and, after continued employment, its analgesic properties are lost.—*Thereapeutic Gazette.*

